# Gastrointestinal: Distal esophageal squamous papillomatosis in a healthy 47‐year‐old man

**DOI:** 10.1002/jgh3.12973

**Published:** 2023-09-25

**Authors:** Gurtej Singh, Kostas G Brooks

**Affiliations:** ^1^ Department of Gastroenterology and Hepatology Bankstown‐Lidcombe Hospital Sydney New South Wales Australia; ^2^ South West Sydney Clinical School University of New South Wales Sydney New South Wales Australia

**Keywords:** esophageal disorder, esophagus, human papilloma virus, squamous papillomatosis, upper gastrointestinal endoscopy

## Abstract

A 47‐year‐old man with a background history of gastroesophageal reflux disease (GERD) and seasonal asthma underwent a gastroscopy for further investigation. Endoscopy revealed numerous polypoid lesions diffusely distributed in the lower third of the esophagus, with histology revealing squamous papilloma with occasional intraepithelial lymphocytes. The diagnosis was esophageal squamous papillomatosis (ESP), which is a rare condition characterized by exophytic and circumferential projections with friable mucosa diffusely spread through the esophagus with unclear etiology and malignancy risk.

## Graphical abstract

Esophageal squamous papillomatosis in an otherwise healthy 47‐year‐old man seen on endoscopy as part of investigation for reflux symptoms.

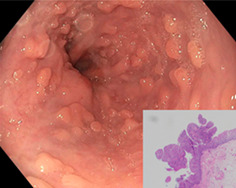

## Introduction

Esophageal squamous papillomatosis (ESP) is a rare condition characterized by well‐defined and exophytic circumferential projections, with friable mucosa diffusely spread through the esophagus. The incidence of singular or limited squamous esophageal papillomas is proposed to be between 0.01% and 0.04% of the population,^1^, ^2^ while diffuse ESP is exceedingly rare with only 17 cases reported to our knowledge.^3^, ^4^, ^5^, ^6^, ^7^, ^8^, ^9^, ^10^, ^11^, ^12^, ^13^, ^14^, ^15^, ^16^, ^17^, ^18^, ^19^

## Case report

**Figure 1:**
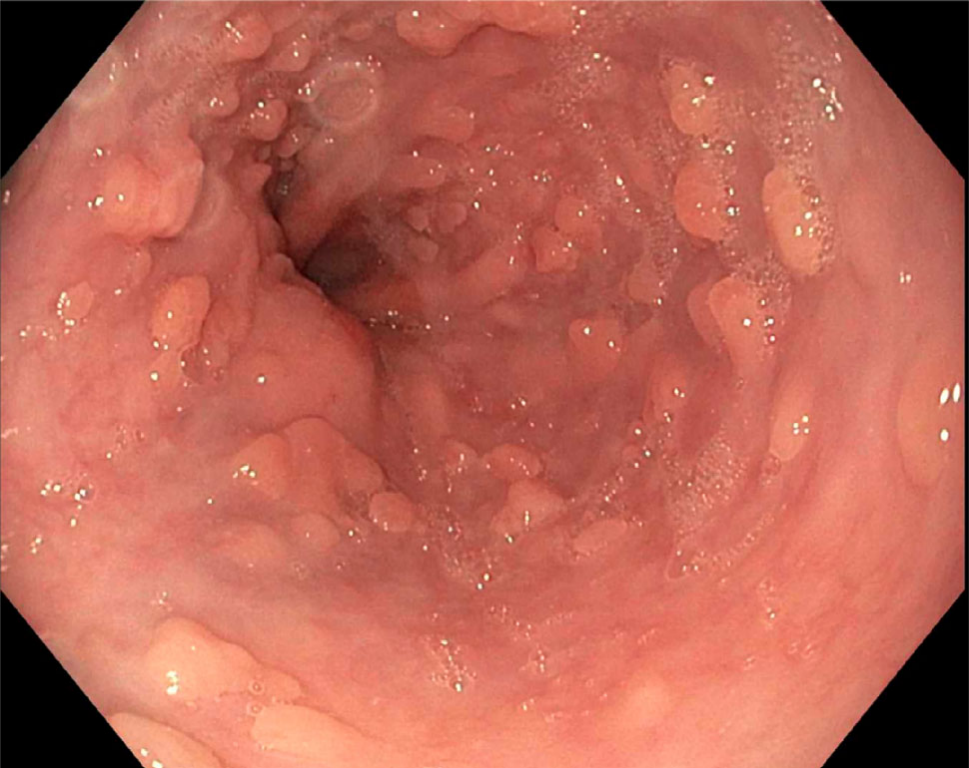
Endoscopic image of the distal esophagus showing diffuse squamous papillomas in a circumferential distribution.

**Figure 2:**
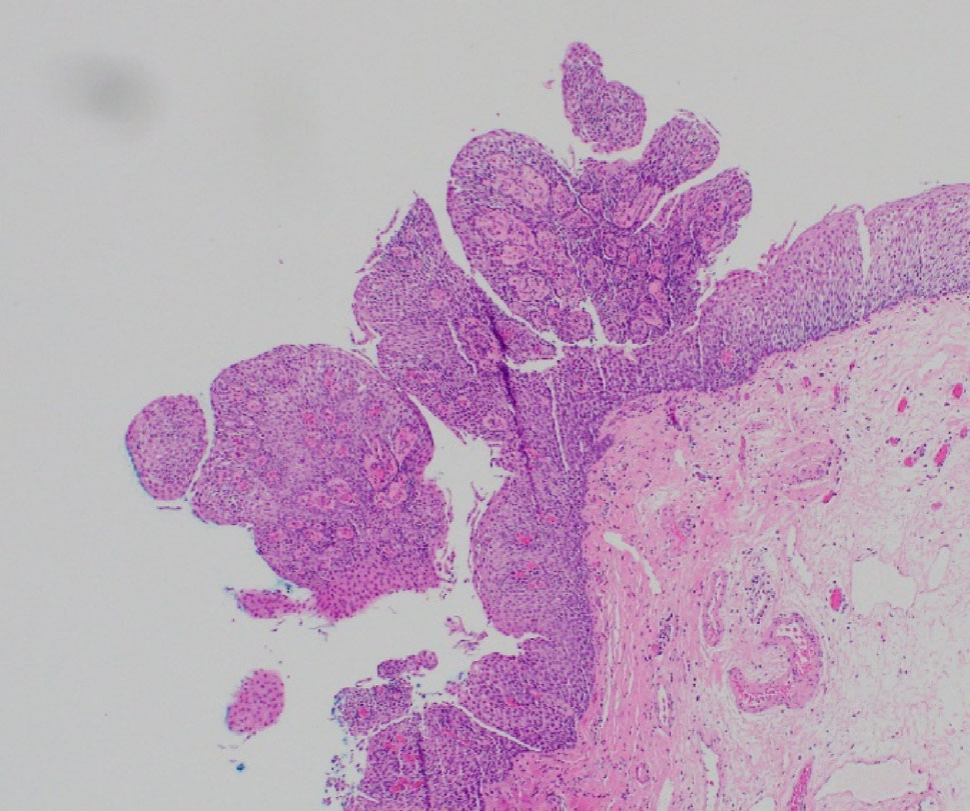
Biopsy of the lesions showing papillomas with squamous epithelium and sparse lymphocyte infiltration.

A 47‐year‐old man with a background history of gastroesophageal reflux disease (GERD) and seasonal asthma underwent a gastroscopy for further investigation. There was no history of regurgitation, dysphagia, weight loss, or melena. There was no relevant past medical history, and laboratory tests were unremarkable. Cross‐sectional imaging with computed tomography of the abdomen and pelvis revealed no significant anatomical findings. Endoscopy revealed numerous polypoid lesions diffusely distributed in the lower third of the esophagus with sparing of the mid and proximal esophagus (Fig. 1). Endoscopic resection of a lesion was performed, which showed squamous papilloma with occasional intraepithelial lymphocytes. Additionally, there was no viral cytopathic changes or epithelial atypia. No vacuolization or inclusion bodies were seen. Importantly, no evidence of dysplasia or malignancy was present (Fig. 2). The diagnosis was ESP.

## Discussion

ESP is a rare condition characterized by well‐defined, exophytic, circumferential projections, with friable mucosa diffusely spread through the esophagus. The pathogenesis of ESP remains unclear. Two major theories are suggested. The first is that mucosal irritation caused by mechanical and chemical irritants results in cellular damage and then hyper‐regeneration through damage‐repair mechanisms. Approximately 70% of singular esophageal papillomas are located in the distal third of the esophagus and have been associated with reflux, esophagitis, prolonged nasogastric intubation, and nitrosamine exposure, supporting this theory.^2^, ^20^, ^21^, ^22^, ^23^ The second proposed pathophysiological mechanism involves human papilloma virus (HPV). In Italy and Hungry, HPV infection has been found in 26–41% of singular esophageal squamous papillomas,^2^ although in other studies, HPV has been found only in ~5% of esophageal papillomas.^24^ Both pathological mechanisms may be synergistic.

The clinical progression of singular lesions has been shown to be highly variable, ranging from regression to progression to squamous cell carcinoma.^25^ As such, it is difficult to ascertain whether singular papillomas represent premalignant lesions. Conversely, diffuse esophageal papillomatosis is thought to have a high malignant potential, with squamous cell carcinomas reported in nearly 50% of published cases.^4^, ^11^, ^13^, ^14^, ^15^, ^17^, ^18^, ^19^ Consequently, treatment of ESP is of upmost importance. Unfortunately, the paucity of published cases means decisions regarding optimum treatments and surveillance are unclear. Previous published reports have used Nd:YAG laser ablation, endoscopic resection, radio‐ablation, and cryotherapy to varying levels success.^4^, ^7^, ^8^, ^10^, ^13^, ^16^, ^26^ Continual research pertaining to the treatment and surveillance of this rare condition is needed to guide future therapeutic options and recommendations.

### Patient consent statement

Informed consent was obtained from the patient for the use of data and images for this manuscript.
